# Emerging and Re-emerging Infectious Diseases in the WHO Eastern Mediterranean Region, 2001-2018

**DOI:** 10.34172/ijhpm.2021.13

**Published:** 2021-03-06

**Authors:** Ehsan Mostafavi, Abdolmajid Ghasemian, Abubakar Abdinasir, Seyed Alireza Nematollahi Mahani, Salman Rawaf, Mostafa Salehi Vaziri, Mohammad Mahdi Gouya, Tran Minh Nhu Nguyen, Salah Al Awaidy, Lubna Al Ariqi, Md. Mazharul Islam, Elmoubasher Abu Baker Abd Farag, Majdouline Obtel, Peter Omondi Mala, Ghassan M. Matar, Rana Jawad Asghar, Amal Barakat, Mohammad Nadir Sahak, Mariam Abdulmonem Mansouri, Alexandra Swaka

**Affiliations:** ^1^Department of Epidemiology and Biostatistics, Research Centre for Emerging and Re-emerging Infectious Diseases, Pasteur Institute of Iran, Tehran, Iran.; ^2^Infectious Hazards Management, World Health Organization, Eastern Mediterranean Regional Office, Cairo, Egypt.; ^3^Department of Primary Care and Public Health, School of Public Health, Faculty of Medicine, Imperial College, London, UK.; ^4^Department of Arboviruses and Viral Hemorrhagic Fevers, Research Centre for Emerging and Re-emerging Infectious Diseases, Pasteur Institute of Iran, Tehran, Iran.; ^5^Centre for Communicable Disease Control, Ministry of Health and Medical Education, Tehran, Iran.; ^6^Office of Health Affairs, Ministry of Health, Muscat, Oman.; ^7^Department of Animal Resources, Ministry of Municipality and Environment, Doha, Qatar.; ^8^School of Laboratory Medicine and Medical Sciences, College of Health Sciences, University of KwaZulu Natal, Durban, South Africa.; ^9^Ministry of Public Health, Doha, Qatar; ^10^Laboratory of Community Medicine, Preventive Medicine and Hygiene, Public Health Department, Faculty of Medicine and Pharmacy, Mohammed V University, Rabat, Morocco.; ^11^Laboratory of Epidemiology, Biostatistics and Clinical Research, Public Health Department, Faculty of Medicine and Pharmacy, Mohammed V University, Rabat, Morocco.; ^12^Department of Experimental Pathology, Immunology and Microbiology Center for Infectious Diseases Research, American University of Beirut & Medical Center, Beirut, Lebanon.; ^13^University of Nebraska Medical Center, Omaha, NE, USA.; ^14^Global Health Strategists & Implementers (GHSI), Islamabad, Pakistan.; ^15^Infectious Hazard Management Department, World Health Organization, Kabul, Afghanistan.; ^16^Communicable Diseases Control Department, Public Health Directorate Unit, Ministry of Health, Kuwait City, Kuwait.; ^17^Centre for Public Health, Queen’s University Belfast, Belfast, UK.

**Keywords:** Neglected Tropical Diseases, Emerging Infectious Diseases, Zoonosis, MERS-CoV, CCHF, Eastern Mediterranean Region

## Abstract

**Background:** Countries in the World Health Organization (WHO) Eastern Mediterranean Region (EMR) are predisposed to highly contagious, severe and fatal, emerging infectious diseases (EIDs), and re-emerging infectious diseases (RIDs). This paper reviews the epidemiological situation of EIDs and RIDs of global concern in the EMR between 2001 and 2018.

**Methods:** To do a narrative review, a complete list of studies in the field was we prepared following a systematic search approach. Studies that were purposively reviewed were identified to summarize the epidemiological situation of each targeted disease. A comprehensive search of all published studies on EIDs and RIDs between 2001 and 2018 was carried out through search engines including Medline, Web of Science, Scopus, Google Scholar, and ScienceDirect.

**Results:** Leishmaniasis, hepatitis A virus (HAV) and hepatitis E virus (HEV) are reported from all countries in the region. Chikungunya, Crimean Congo hemorrhagic fever (CCHF), dengue fever, and H5N1 have been increasing in number, frequency, and expanding in their geographic distribution. Middle East respiratory syndrome (MERS), which was reported in this region in 2012 is still a public health concern. There are challenges to control cholera, diphtheria, leishmaniasis, measles, and poliomyelitis in some of the countries. Moreover, Alkhurma hemorrhagic fever (AHF), and Rift Valley fever (RVF) are limited to some countries in the region. Also, there is little information about the real situation of the plague, Q fever, and tularemia.

**Conclusion:** EIDs and RIDs are prevalent in most countries in the region and could further spread within the region. It is crucial to improve regional capacities and capabilities in preventing and responding to disease outbreaks with adequate resources and expertise.

## Introduction

 Emerging infectious diseases (EIDs) are those that have recently appeared within a population or those whose incidence or geographic range is rapidly increasing or threatens to increase shortly. Re-emerging infectious diseases (RIDs) are those which were previously controlled, but have again risen to be a significant health threat.^1,2^ The emergence of high-threat pathogenic diseases has increased in recent years globally.^2^ Almost 75% of recently emerged diseases afflicting humans have a zoonotic origin.^2^

 Many countries in the World Health Organization (WHO) Eastern Mediterranean Region (EMR) are affected directly or indirectly by acute and protracted humanitarian emergencies, which have led to an unusually high number of internally displaced people and refugees living in overcrowded, overburdened camps, with little or no access to basic social and healthcare services.^3^ Many factors are contributing to the emergence or re-emergence of high-threat pathogenic diseases including pathogen’s adaptation or resistance, host behavior such as migration, international travel, human-animal interaction, poverty, climate change, and industrial and economic development.^4^

 WHO EMR includes countries from North Africa to southwest Asia, with a total population of 670 million, Pakistan, Egypt, and Iran with about 200, 105, and 85 million people are respectively the most populated countries in this region.^5^

 EIDs and RIDs occur as a result of interconnection among social, economic, biological, technological, and ecological factors. A high percentage of EMR populations live in poverty with the regional average gross domestic product per capita lagging behind the global average,^6^ with significance in Somalia, Afghanistan, Yemen, Syria, and Sudan^7^; thus, further predisposing the region to EIDs and RIDs. Different climate and living conditions lead to the spread of various diseases,^8^ with ecological changes playing an important role in the re-emergence of infectious diseases.^9^ The periodic mass gathering of pilgrims in the EMR, namely to Saudi Arabia and Iraq, can also be a threat for outbreaks of EIDs and RIDs.^10^ Increased conflict and political instability in the region that lead to large population movement are other major causes of elevating the risk of spreading various diseases (eg, diphtheria, cholera, and leishmaniasis).^11^

 Recent outbreaks of EIDs and RIDs in the EMR include Crimean Congo hemorrhagic fever (CCHF) in Afghanistan^12^, chikungunya in Pakistan and Sudan,^13,14^ cholera in Somalia, and Yemen,^15^ diphtheria in Pakistan and Yemen,^16,17^ influenza H5N1 in Egypt,^18^ leishmaniasis in Pakistan, Syria and Afghanistan,^19-21^ measles in Pakistan and Afghanistan,^16,22^ Middle East respiratory syndrome (MERS) in Arabian Peninsula,^23-25^ plague in Afghanistan,^26^ polio in Afghanistan and Pakistan,^27,28^ and Q fever in Afghanistan and Iraq.^29,30^ The magnitude of many of EIDs and RIDs has not been currently verified in some EMR countries. The first step in forecasting, preparing, and control of these diseases is to ascertain its existence and frequency. This paper aims to review the epidemiological situation of the EIDs and RIDs in the EMR between 2001 and 2018.

## Methods

 This paper is a narrative review. An exhaustive list of emerging and RIDs was identified through a literature search. The preliminary list was then shared with EMR 17 experts using Delphi method. The experts covered a range of specialities, including microbiology, epidemiology, and clinical infectious disease. Based on the expert’s opinion, a final list of infectious diseases of global concern in EMR was identified and approved.

 A review about the epidemiological situation of the EIDs and RIDs in the EMR was conducted. A complete list of studies in the field was prepared following a systematic search approach. Studies that were purposively reviewed were identified to summarize the epidemiological situation of each targeted infectious disease in different EMR countries. Accessible electronic and hand-search of grey literature across all countries of the region were searched.

###  Systematic Search of Literature

 Electronic databases were carefully searched using Medical Subject Headings terms and keywords, by the name of each of the targeted diseases or their causative agent and the name of each country in the region to extract the diseases reported between 2001 and 2018. The extensive databases included: Google Scholar, Midline, Web of Science, ScienceDirect, Scopus, Medline. We also searched grey literature via a general internet search, Web of Science, Weekly Epidemiological Monitor, WHO/EMRO, situation updates, annual reports, and report publications by the WHO, the official WHO website, and ProMED. The reference lists of all relevant articles were hand-searched to identify further any additional studies that may not have been captured by extensive searches.

###  Screening of Potentially Relevant Citation

 Two investigators assessed titles and abstracts for relevance to the key questions using prespecified inclusion and exclusion criteria. Full-text articles identified by either investigator as potentially relevant were retrieved for further review and examined by two investigators independently.

 All outcomes were adequately covered in the EndNote^®^ reference management software (version X5, Thomson Reuters, Philadelphia, PA, USA).

###  Inclusion Criteria

 Eligible subjects invariably had to satisfy the following inclusion criteria sufficiently including, study designs: Published papers from 2001 and 2018 that reported the epidemiological situation of the targeted diseases in the region were included. The ultimate goal was to typically identify related systematic reviews, review articles, case reports and original articles and it was limited to Arabic and English language publications.

###  Quality Assessment

 A narrative review was based on high-quality evidence. The methodological quality of studies that were a candidate for data extraction for this narrative review was subjectively appraised by two investigators, considering the following criteria: (*a*) No evidence of selection bias, (*b*) Proper sample size, and (*c*) Negligible publication bias (for systematic reviews).

**Figure 1:**
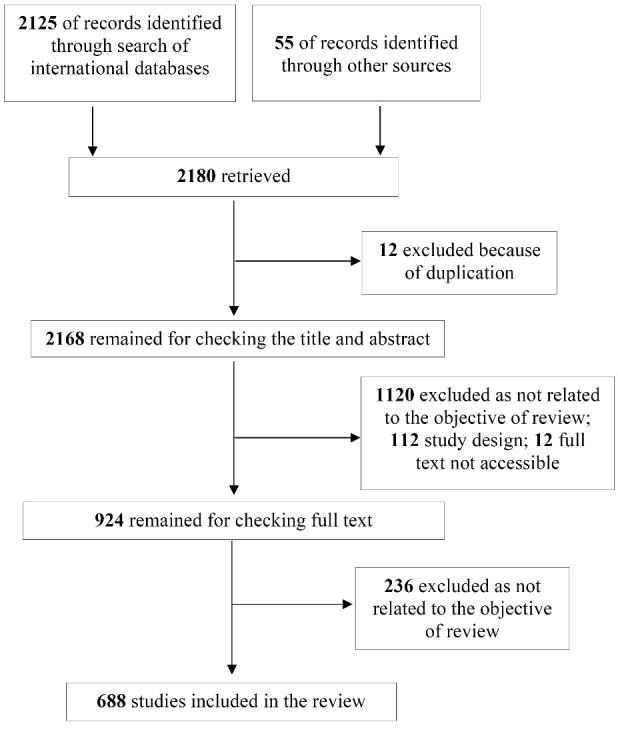
A Flow Chart Depicts the Stages of Retrieving References, Checking Eligibility Criteria, and Including the Final Articles Into The Review.

 Disagreements were resolved between the two investigators by discussion. A flowchart of the study selection process is illustrated in Figure 1.

###  Data Abstraction and Analysis

 Extracted data were abstracted into a customized Excel spreadsheet, extensive database by one investigator and verified by a second investigator. All related papers found were carefully reviewed and succinctly summarized for potential inclusion in this report. The included diseases are listed in three groups (viral, bacterial, and parasitic EIDs and RIDs) alphabetically by common name.

## Results

###  Viral Emerging and Re-emerging Infectious Diseases 

**Acute hepatitis A and E**: Except for a few published articles and outbreak reports, very limited data are available about the prevalence of hepatitis A virus (HAV) and hepatitis E virus (HEV) from countries in the EMR.^31^ HAV and HEV are reported in high frequency in some studies in Tunisia (84%),^32^ Yemen (86%),^33^ Iran (86%), Iraq (96%),^34^ Egypt (100%),^35^ and Libya (100%).^36^ Morocco is an intermediate endemic area for the HAV infection.^37^ Most rural areas have high anti-HAV antibody prevalence due to consumption of sewage-contaminated water and use of indoor dry pits.^38^ Several outbreaks of HAV infection have erupted among tourists from European countries.^39^ There were major HAV outbreaks in Syria and Lebanon in 2013 and 2014, concurrent with the Syrian crisis and influx of refugees.^40,41^

 In Iran, the frequency of HEV varied from 2.3% to over 40%,^42^ and have reported 20% in United Arab Emirates (UAE) in mothers,^43^ 19.4% in Iraq in blood donors,^34^ 13% in Egypt in workers,^44^ 10% in Yemen,^33^ 3% in Pakistan,^45^ and 0.3% in Saudi Arabia.^46^ According to reports from Pakistan, HAV and HEV are responsible for more than 19% and 12% of all newly diagnosed cases of viral hepatitis, respectively.^31^

**Alkhurma hemorrhagic fever (AHF)**: AHF is a zoonotic viral disease that emerged in 1995 in Saudi Arabia.^47^ In the initial stages of the disease discovery, high mortality rates of up to 25% were reported.^48^ As the disease became better known and recognizable, identification of subclinical infections lowered the mortality rates to about 1.3%.^49^ There is still a knowledge gap regarding the transmission pathways of the disease.^50^

 AHF has been reported from Saudi Arabia and Egypt.^51,52^ Seropositive cases and AHF virus-infected *Amblyomma lepidum* ticks have been reported in Djibouti.^53^

**Avian influenza (H5N1)**: The virus was reported in 16 countries and is expected to expand its range further. In 2006, H5N1 spread rapidly through the EMR with large non-human (mostly avian) outbreaks in Afghanistan, Djibouti, Egypt, Iran, Iraq, Jordan, occupied Palestinian territories, Pakistan, and Sudan. Since then the H5N1 has become endemic in Egyptian poultry.^54^ The circulation of the virus was confirmed in Saudi Arabia, Egypt, and Libya.^55,56^ Since 2006, Human cases of H5N1 were reported only from Egypt, Iraq, Djibouti, and Pakistan.^57^ However, Egypt has been the most affected country in the region where the disease has remained endemic, with frequent epizootic cases in addition to 356 reported human cases, including 121 deaths since October 2016, the highest number of H5N1 human cases (42%, 356/854) in the world.^58,59^ A recent analysis demonstrated that the most important environmental predictors for the spread of the disease in the Middle East are the environmental temperature during the warmest quarter in correlation with high transmission rates within the livestock system with Egypt, Kuwait, Saudi Arabia, and Sudan.^60^ While nearly all cases in EMR have been associated with contact with infected birds, human-to-human transmission of H5N1 has also been indicated in Djibouti, Iraq, and Pakistan, but limited.^61^

**Chikungunya**: Outbreaks of Chikungunya have only been documented in Djibouti, Pakistan, Saudi Arabia, Somalia, Sudan, and Yemen. Imported cases are also reported from Oman.^14,62^ Nevertheless, serological studies in other countries such as Egypt, Iraq, Iran, and Kuwait, have reported seropositivity cases.^63^ The earliest description of the disease in the EMR goes back to 1658 in Egypt. However, the disease presence was confirmed in 2011 in Yemen after a major outbreak with over 15 000 suspected cases.^64^

 Similarly, in Pakistan, the serological evidence of disease was first identified in 1983, but no further cases had been identified until 2011. Since then, the total number of infected cases drastically increased to 8330 reported cases in 2017 compared to 405 cases found in the previous year.^65,66^ Considering the presence of the competent vectors and international travel of viremic patients between Eastern Mediterranean countries, there seems to be a hidden crisis with a high possibility of transmission and spread of the disease into neighboring chikungunya virus-free countries.^63^

**CCHF**: So far, human cases of CCHF have been reported from 10 out of 22 countries in EM region including Afghanistan, Iran, Iraq, Oman, Pakistan, Palestine, Saudi Arabia, Sudan, Tunisia, and UAE.^67-70^ Iran, Pakistan, and Afghanistan are countries reporting 50 or more CCHF cases per year.^71-73^ The CCHF virus genome has been identified in ticksin Morocco and Syria.^74,75^ Moreover, serological studies of livestock have identified the disease in Egypt and Somalia.^67^ In Oman, from 2011-2017, the CCHF patients has steadily increased, the highest cases were reported in 2015.^76^

**Dengue**: There is limited information about the situation of the disease in EMR. Outbreaks of dengue have occurred in Djibouti, Oman, Pakistan, Saudi Arabia, Somalia, Sudan, Yemen, and Egypt.^77-79^ Additionally, serological investigations have shown seroprevalence of dengue in Afghanistan, Iran, Kuwait, Lebanon, Qatar, and Syria.^80^ The main mosquito vectors for dengue virus, *Aedes aegypti* and* Aedes albopictus*, have been reported from 15 countries in the region including Afghanistan, Djibouti, Egypt, Iran, Jordan, Lebanon, Oman, Pakistan, Palestine, Saudi Arabia, Somalia, Sudan, Syria, Tunisia, and Yemen.^81-84^ However, little is known about the presence of infected vectors in other EM countries, due to the inadequate entomological surveillance systems in most of these countries. In 2017, Pakistan, Egypt, and Sudan reported 125 316, 245, and 139 dengue cases, respectively.^66^

 A total of 6777 suspected dengue cases were reported in 2015 in Yemen after the civil war, which began in 2015, causing widespread destruction of the infrastructure and therefore enabling dengue to become endemic in this country.^85^

**Measles**: In 1997, all EMR countries adopted a resolution to eliminate measles by 2010. This effort resulted in a 93% reduction in mortality from measles between 2000 and 2008. Despite significant progress, the goal was not reached by 2010, and the date was revised to 2015 and then again to 2020.^86^ Unlike the predictions, the total number of measles cases increased from over 12 000 cases in 2008 to over 36 000 in 2012.^87^ Between 2013 and 2018, 144 966 cases of Measles were reported in the region.^88^ All countries in the region have moved to case-based measles surveillance with laboratory confirmation implementing nationwide surveillance and two countries (Somalia and Sudan) implementing sentinel surveillance. Countries affected by war, such as Syria, Yemen, Iraq, Afghanistan, and Lebanon reported high numbers of measles cases, for example during 2013-2016, 7000 cases of measles were reported in Syria, and 5773 cases were reported from Yemen during 2012-2017.^89-91^ Pakistan also reported a high number of measles patients due to low vaccination coverage.^92^ Djibouti, Somalia, and Sudan are also countries in the region with high incidence.^87^ Bahrain, Egypt, Iran, Jordan, Morocco, Palestine, and Tunisia have progressed drastically and reported a very low incidence of endemic measles.^93^ Nevertheless, the surge of disease in countries affected by armed conflicts and political instability shows how easily it can return, especially with the low immunization coverage.

**MERS**: MERS was first reported in Saudi Arabia in 2012.^94^ In EMR, 12 countries (Bahrain, Egypt, Iran, Jordan, Kuwait, Lebanon, Oman, Qatar, Saudi Arabia, Tunisia, UAE, and Yemen) have reported cases of MERS.^95-98^ By the end of 2018, a total of 2279 confirmed cases were reported globally with the majority of cases in Saudi Arabia (1901 laboratory-confirmed cases, including 732 related deaths with a case–fatality ratio of 38.5%).^99^ Most of the primary MERS cases outside Arabian Peninsula were linked with either by travel or residence in the countries of Arabian Peninsula.^100^ Transboundary movement of humans and camels among the Arab countries could be a source of MERS coronavirus (MERS-CoV) transmission.^101^ Clusters of MERS secondary cases were reported either in hospital or family settings in Saudi Arabia, UAE, and Iran.^102-104^ In Saudi Arabia, hospital outbreaks in 2015-2018 resulted in 334 cases and 102 associated deaths, and approximately 30% of these reported cases were healthcare workers.^105^ In Qatar, MERS pattern were mostly sporadic within the primary cases. It may be due to Qatar One Health approach to challenge MERS-CoV, and infection protection and control system in healthcare settings.^106^

 Egypt, Qatar, UAE, Oman, Morocco, and Sudan, among other countries within the EM region carried out surveillance studies on dromedary camels. Results obtained revealed high seropositivity in Egypt (84.5%) and Qatar (59% and 79%); besides, MERS-CoV genetic material was identified in 6.57%, 3.8%, and 1.6% of sampled camels from Oman, Egypt and UAE, respectively.^25,107-112^

**Rabies**: Currently, zoonotic rabies remains mostly in dogs as the principal reservoir in the Middle East.^113^ Incidence from foxes was reported in UAE and Oman, and from wolves in Syria.^114^ Rabies is reported endemic in Egypt, Iran, Iraq, Pakistan, Sudan, Tunisia, Morocco, Syria, Yemen, Jordan, Oman, Palestine, Lebanon, and Saudi Arabia.^115,116^ The UAE, Bahrain, and Kuwait are the only countries in the region that are considered rabies-free.^115^ Libya, Somalia, and Djibouti have no information available about rabies in humans or animals.^116^ The disease rate in animals has sharply increased in Lebanon from 2010-2016 coinciding with the beginning of Syrian conflict.^117^

**Rift Valley fever (RVF)**: Outbreaks of RVF have been documented in the Arabian Peninsula in Yemen and Saudi Arabia (2000, 516 cases with 87 deaths),^118-120^ Egypt (2003, 148 cases with 27 deaths), Somalia (2006-2007, 114 cases with 51 deaths) and Sudan (2007-2008, 738 cases with 230 deaths).^121,122^ In 2017, seropositive livestock was reported in western Iran.^123^ With the virus showing high competence for a wide range of mosquitoes, the WHO predicts future outbreaks in Egypt, Sudan, Morocco, Saudi Arabia, and Yemen.^124^

**Sandfly fever**: Although research has demonstrated the circulation of sandfly virus in Afghanistan, Egypt, Iran, Iraq, Morocco, Pakistan, Palestine, Tunisia, Saudi Arabia, Somalia, and Sudan,^125-127^ little is known about the epidemiology of sandfly fever in the EMR. Dashli virus belonging to the Sicilian serogroup and two cases of severe encephalitis caused by sandfly fever virus were reported from Iran and occupied Palestine.^128-130^ Furthermore, antibodies against sandfly fever virus have been detected in Iran.^131^ In 2010-2011, a serological investigation in Djibouti indicated the circulation of Toscana-related viruses.^132^ In 2007, an outbreak of sandfly fever was reported from Lebanon with 700 cases.^133^ In 2017, cases were also reported from Afghanistan.^125^

**West Nile fever (WNF)**: Human seropositive cases of WNF have been reported in Afghanistan, Djibouti, Egypt, Iran, Iraq, Jordan, Lebanon, Libya, Morocco, Pakistan, Sudan, Tunisia, and Yemen.^134^ WNF infection in Culex mosquitoes was demonstrated in Djibouti, Egypt, Iran, and Tunisia.^134^ This indicates the widespread circulation of WNF virus in the EMR and underlines the requirement for integrated surveillance programs. In 2018, an outbreak of WNF with 377 suspected cases, out of which 49 cases were laboratory-confirmed, was reported from Tunisia.^135^

**Poliomyelitis**: In April 2013, a case of wild poliovirus was detected in Somalia, which quickly spread, affecting 194 people by the end of 2013. The long-term political instability in Iraq and Syria means they are key at-risk countries for re-emergence of polio.^136^ Syria had a disease outbreak in 2014 that was closely associated with the virus originating from Pakistan. In 2017, Syria was affected by circulating vaccine-derived poliovirus.^137^ The last reported polio cases in Somalia were five new cases in 2014.^138^ In 2017, 22 cases of wild poliovirus were found in Afghanistan and Pakistan, of which increased to 33 cases in 2018.^28,66^ Today the disease is only seen in Afghanistan, Pakistan, and Nigeria where vaccination was not fully covered.^139^ As the virus remains present in the EMR, all countries in the region are still at high risk for re-emergence of the disease.

###  Bacterial Emerging and Re-emerging Infectious Diseases 

**Cholera**: Cholera is a disease resulting from poor sanitation and living conditions. The disease is widespread across the EMR. Between 2010 and 2016, Iran, Afghanistan, Pakistan, Yemen, Iraq, and Somalia reported the disease.^140-142^ In 2017, the largest outbreak of cholera in regional history was witnessed in Yemen with 1.3 million cases, and over 2500 deaths were reported by the end of 2018.^143^ The second most affected country in the EMR is Somalia reporting 75 414 cases and 1007 associated deaths since the outbreak started in 2017. Other countries in the region that reported imported cases of cholera in 2017 were Qatar (5), Saudi Arabia (5), UAE (12), and Iran (625).^144^ There are concerns about the rise of antibiotic resistance against cholera, due to mobile genetic elements in Yemen.^145^

**Diphtheria**: Pakistan (930 cases), Iran (631 cases), and Sudan (225 cases) are among the list of countries with the highest prevalence of diphtheria between 2010 and 2017.^146,147^ Between 2001 and 2018, cases of diphtheria are also reported to the WHO from Afghanistan, Iraq, Lebanon, Qatar, Saudi Arabia, Somalia, and Yemen,^17^ and no cases of diphtheria were reported from Bahrain, Djibouti, Egypt, UAE, Jordan, Kuwait, Morocco, Oman, and Tunisia.^147^ Furthermore, countries such as Libya and Syria, which have been involved in civil wars, have not been evaluated for disease presence. In Yemen, due to ongoing war resulting in disruption of the healthcare system and lower vaccination coverage, a total of 2609 cases of the disease were reported by the end of 2018,^17^ even though the last outbreak in Yemen was in 1982. These outbreaks represent the great potential of diphtheria to re-emerge in disease-free areas and become endemic.^146^

**Meningococcal meningitis**: The majority of the cases of meningococcal meningitis occur in sub-Saharan African countries. In EMR, Sudan is the only country at high risk of the disease; however, other countries, mainly North African countries, are also at risk.^148^ Sudan has received a high burden of disease, experiencing several large outbreaks in 2013.^148,149^ In 2016, the meningitis A vaccination was introduced into Sudan’s routine immunization program.^150^ In 2017, an outbreak of meningitis was reported in Yemen with 2982 cases and 37 deaths.^66^

 Although meningococcal meningitis remains an important cause of endemic and epidemic disease across the region, published epidemiological data is fragmented and limited, and the use of vaccines has helped minimize the prevalence of meningococcal meningitis.^151,152^ The meningitis belt is highly connected by Muslim pilgrims taking part in the annual Hajj ceremony, gathering in Saudi Arabia, as the congestion of people promotes increased carrier rates of meningitis.^148^ The Global Meningococcal Initiative has recommended that EMR countries should mandate vaccination; especially those in which the Hajj is obligatory.

**Plague**: An outbreak of plague was reported in Afghanistan in 2007, and 17 out of 83 presumed cases became fatal.^26^ In 2009, 3 cases of plague were reported from Libya after 25 years of disease absence.^153^ Two outbreaks occurred in the years 2009 and 2011 in a coastal town in Libya.^154,155^ Western Iran has remained as endemic areas for the plague. A study in 2011 in this region detected *Yersinia pestis *in 1.02% of trapped rodents and 3.42% of dogs.^156^ The circulation of plague among domestic and wild animals in the region indicates possible re-emergence of human plague outbreaks.^157^

**Q Fever**: All countries in the region have detected the disease in humans except Bahrain, Djibouti, Palestine, and UAE.^30,158-163^ There was an outbreak of the disease among US military soldiers in Western Iraq in 2005.^29^ A similar outbreak was reported among British soldiers in Afghanistan in 2008.^160^ A systematic review reported 19% of Q fever seroprevalence in North Africa.^162^

**Tularemia**: Most of the EMR countries have not reported the disease in recent years due to lack of laboratory facilities and healthcare workers’ suboptimal awareness of the disease.^164,165^ Iran is the only country in the region, reporting the disease and contaminated water is the main source of infection.^164,166^ However, studies on rodents and hares in Iran,^167^ on ticks in Yemen,^168^ and ticks and abattoir workers in Egypt found samples positive for *Francisella tularensis.*^169^

###  Parasitic Emerging and Re-emerging Infectious Diseases 

**Leishmaniasis**: All EM countries are reporting the cutaneous and mucocutaneous forms, though the visceral form has limited reports in this region.^170^ Six of the ten countries with the highest reported cutaneous leishmaniasis in the world are located in the EMR including Syria, Afghanistan, Iraq, Iran and Pakistan, and Tunisia.^171^ The incidence of leishmaniasis has generally decreased in EMR; however, this region still accounts for 70% of all leishmaniasis cases across the world.^172^ The zoonotic form of visceral leishmaniasis is endemic in Iran.^173^ In Iraq, most leishmaniasis cases are wet type cutaneous form.^174^ Studies in Yemen have found *Leishmania tropica*as the main causative agent of leishmaniasis.^175^*Leishmania tropica*is more prevalent in Morocco with a rate of 30%-40% in several districts.^176^ In 2016 and 2017, outbreaks of leishmaniasis were reported in Pakistan.^177^ In 2017 in a study in Saudi Arabia, 8.3% of studied individuals were positive for cutaneous leishmaniasis.^178^

 In recent years, the incidence of cutaneous leishmaniasis has increased in Syria.^179,180^ Lebanon, Iraq, and Egypt are affected due to Syrian refugee migration. *Leishmania tropica*is detected in 85% of the Syrian refugee patients in Lebanon.^11^ Eastern Libya, similar to Syria and Yemen, has reported the outbreaks of cutaneous leishmaniasis.^181,182^

## Discussion

**Figure 2:**
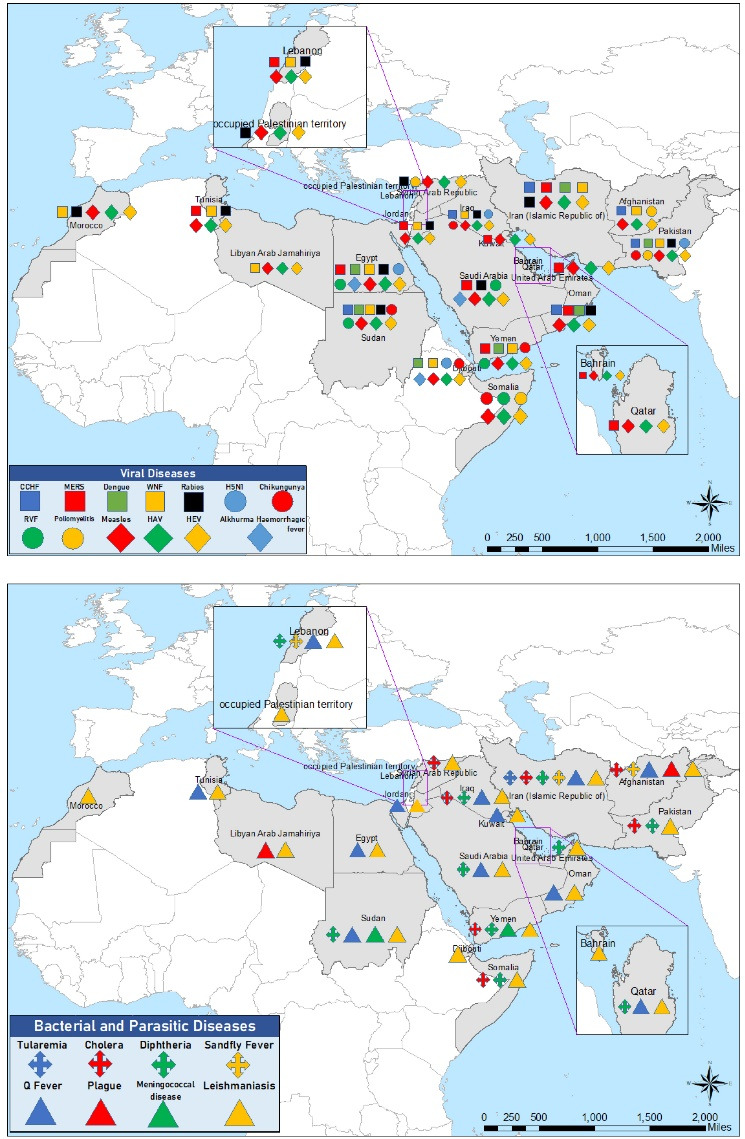
EIDs in Humans Reported in the EMR, 2001-2018. Abbreviations: EMR, Eastern Mediterranean Region; EIDs, emerging infectious diseases. Abbreviations: CCHF, Crimean-Congo hemorrhagic fever; MERS-CoV, Middle East respiratory syndrome coronavirus; RVF: Rift Valley fever; WNF, West Nile fever; HAV, hepatitis A virus; HEV, hepatitis E virus.

 EMR is a hotspot for EIDs and RIDs. Although it’s difficult to compare the extend and burden of EID and RIDs in this region with other regions, the number of outbreaks caused by emerging and re-emerging infectious pathogens has increased in the past two decades in this region, greatly affecting social and economic development. The region is especially susceptible to outbreaks of these high-threat pathogens due to the presence of various humanitarian emergencies, fragile health systems, internal conflicts, lack of accurate data, fragile ecosystems, and increased population movement. In recent years, the frequency, duration, and scale of disease outbreaks have escalated for most of these diseases. Many disease outbreaks have been detected and managed in the region such as MERS in the Arabian Peninsula, cholera in Iraq, Somalia and Yemen, Avian influenza A (H5N1) in Egypt, CCHF in Afghanistan, Iran and Pakistan, and dengue fever in Yemen, Sudan and Pakistan (Figure 2, and Table 1). There is a need for a better understanding of disease transmission, preparedness for disease emergence, detection of pathogens and vectors, and implementation of high-impact control and interventions for prevention. This is especially difficult considering the weak surveillance systems of many countries due to limited diagnostic capacities and human resources. Extensive training programs on disease surveillance and response to health emergencies are needed now more than ever in this region.

**Table 1:** Some Development Criteria and the Reported Outbreaks of the EIDs in human in EMR, 2001-2018^17,183-186^

**Country**	**Population Million (2015)**	**HDI (2017)**	**Life Expectancy**		**GNI Per Capita** **(2011 PPP $) (2017)**	**Reported Outbreaks**
			**Female**	**Male**		
Afghanistan	35.5	LHD	63.2	63.9	0	CCHF (2007-2012, 2016-2018), plague (2007), poliomyelitis (2001-2018), sandfly fever (2017)
Bahrain	1.5	VHHD	80.4	78.8	41 580	MERS-CoV (2016)
Djibouti	0.9	LHD	65.5	62.2	3105	AI (H5N1) (2006), Chikungunya (2011), dengue (2012)
Egypt	84.7	MHD	74.4	68.0	10 355	AI (H5N1) (2006-17), dengue (2015), MERS-CoV (2014), RVF (2003)
Iran	81.2	HHD	79.4	76.5	19 130	CCHF (2001-2018), diphtheria (2010-13), MERS-CoV (2014), tularemia (2017)
Iraq	38.3	MHD	78.8	74.8	17 789	AI (H5N1) (2006), CCHF (2018), diphtheria (2001, 2004, 2009)
Jordan	9.7	HHD	88.1	77.9	8288	MERS-CoV (2012)
Kuwait	4.1	VHHD	87.2	80.7	70 524	MERS-CoV (2013)
Lebanon	6.1	HHD	80.0	75.8	13 378	MERS-CoV (2014, 2017), sandfly fever (2007)
Libya	6.4	HHD	75.0	71.2	11 100	Plague (2009, 2011)
Morocco	35.7	MHD	74.8	73.3	7340	-
Oman	4.6	VHHD	79.5	75.5	36 290	CCHF (2011-2017), dengue (2014, 2018), MERS-CoV (2013-2018)
Pakistan	199.4	MHD	67.5	66.4	5311	AI (H5N1) (2007), CCHF (2001-2018), Chikungunya (2011, 2016-2018), dengue (2012-2018), diphtheria (2012-13, 2017-18), poliomyelitis (2001-2018)
Palestine	4.8	MHD	78.0	75.6	5055	-
Qatar	2.6	VHHD	81.7	79.6	116 818	MERS-CoV (2012-2017)
Saudi Arabia	32.9	VHHD	79.4	75.3	49 680	Diphtheria (2018), MERS-CoV (2012-18)
Somalia	13.8	LHD	57.3	53.7	7480	Chikungunya (2016), diphtheria (2012-13, 2018), poliomyelitis (2013-2014), RVF (2006-2007)
Sudan	40.5	LHD	72.1	68.9	4119	CCHF (2007-11), Chikungunya (2018), dengue (2012-2017), diphtheria (2001, 2008, 2011-2012, 2018), Meningococcal disease (2005-07), RVF (2007-2008)
Syria	18.3	LHD	75.0	65.5	2337	Poliomyelitis (2010, 2014, 2017)
Tunisia	11.5	HHD	80.8	76.2	10 275	MERS-CoV (2013)
UAE	9.4	VHHD	77.0	77.1	67 805	MERS-CoV (2013-2018)
Yemen	28.3	LHD	70.3	66.0	1239	Chikungunya (2010-2011), Dengue (2012, 2016-2018), Diphtheria (2017-2018), Meningococcal disease (2006-2007, 2016-2017), MERS-CoV (2014)

Abbreviations: GNI, gross national income; PPP, purchasing power parity; EMR, Eastern Mediterranean Region; EIDs, emerging infectious diseases; AI, Avian influenza; CCHF, Crimean-Congo hemorrhagic fever; HDI, Human Development Index (broken down into four tiers: VHHD, HHD, MHD, LHD); VHHD, very high human development (0.8-1.0); HHD, high human development (0.7-0.79); MHD, medium human development (0.55-.70); LHD, low human development (below 0.55); MERS-CoV, Middle East respiratory syndrome coronavirus; RVF: Rift Valley fever; UAE, United Arab Emirates.

 National efforts for forecasting and controling EIDs/RIDs in the EM region, need to be complemented by regional approaches. Population movements are among the most important factors that need to be considered when forecasting and controlling EIDs or RIDs. Most of the EIDs and RIDs mentioned in this study are potentially transmissible during religious pilgrimages where people from across the world gather in Saudi Arabia and Iraq during Hajj and Arba’een, subsequently travelling back to their home countries. Likewise, refugees and displaced persons primarily from Iraq, Afghanistan, Syria, and Yemen have been living under fragile health systems that are unable to detect many of the referenced infectious diseases, or even if detected, do not have the resources to control and/or provide treatment. As a result, when refugees from these countries migrate, various diseases inevitably spread across large regions.

 Disasters and wars are further considerations in predicting and monitoring EIDs or RIDs. The ongoing wars in Iraq, Syria, and Yemen have resulted in poor healthcare systems, where previous infrastructures acting as barriers between people and infectious agents have been devastated and therefore contributed to spreading of several diseases. Especially prevalent are food, waterborne, and vector-borne diseases such as cholera, typhoid, dengue fever, leishmaniasis, and plague. As an example, the civil war in Yemen started in March 2015 and has caused more than 2.2 million people to live in shelters with inadequate healthcare services. The damaged infrastructure and poor water and hygiene in the country have created ideal environmental conditions for the spread of infectious diseases leading to an outbreak of dengue fever^85^ and the largest outbreak of cholera in history.^187^

 Several zoonotic diseases, including MERS, tularemia, Q fever, plague, and RVF, are transferred when an individual is in close contact or lives close to competent vectors and disease hosts. Coordination mechanisms between human and animal health sectors are weak in most countries in the region, so the risk of an increase in zoonotic disease transmission and emergence is present.

 Surveillance systems in most countries of the region are not efficient to disseminate the data readily through the routine process. One probable reason can be poor recognition and reporting of some of the referenced diseases such as chikungunya, CCHF, MERS, plague, sandfly fever, tularemia, and WNF by physicians, as cases tend to occur as isolated incidences and sporadically in remote areas.

 The WHO estimates that approximately 45% of infectious diseases occur in lower-income populations. For example, Yemen and Egypt have high percentage of poverty and are predisposed to several infectious diseases.^18,85,145^ Furthermore, miscommunication or absence of communication in the health system and between countries can result in under-reporting that makes prediction of EIDs and RIDs difficult.

 RIDs have recently displayed an upward incidence or prevalence worldwide. For instance, diphtheria outbreaks (in Iran, Pakistan and Sudan), human plague (in Afghanistan and Saudi Arabia), and dengue/dengue hemorrhagic fever (in Pakistan, Egypt, and Sudan) are classified as RIDs in EMR in recent years.^188,189^

 One of the limitations of this paper is that countries differ in their methods of publishing research papers and reliable documents. The availability of data, studies, and papers may not be congruent between countries. Variation in the capacity of individual countries regarding surveillance and laboratory testing may present further limitations; however, the present review has attempted to provide relatively comprehensive information on the situation of the countries of the region by gathering existing data.

###  The Way Forward

 Development and further strengthening of regional and national capacities for surveillance, laboratory diagnosis, prevention, and control of EIDs and RIDs, in line with requirements of the International Health Regulations (2005), is essential. While the WHO continues to provide guidance and support to all countries to improve preparedness, surveillance, and response to EIDs and RIDs in EMR,^183^ considerable gaps and challenges remain to achieve these capacities.^190^ A key strategic consideration for prevention and control of EIDs and RIDs is to establish surveillance capacity for early detection of any occurrences of EIDs or RIDs; as well as the prediction of unexpected occurrence of these high-threat pathogens; and to be able to effectively monitor occurrence patterns of these diseases both nationally and regionally. A wealth of available technology should be implemented in order to help predict, identify, and monitor EIDs or RIDs in the region.^191,192^

 Other key strategic considerations should include effective coordination and collaboration within and between countries in the region, various scientific fields, and public health institutions to facilitate detection and response to EIDs or RIDs. Collaboration between multiple disciplines through the One Health approach for zoonotic diseases, for example, can lead to early detection, effective response, and better health for people, animals and the environment.^193^ According to the ‘One Health Paradigm’ for global health, the emergence of the majority of new human infectious diseases originates from animal reservoirs. This underscores the need for coordinated surveillance to monitor zoonotic diseases among animals^194^ and implementation of preventive One Health Interventions.^106^

 Campaigns to control mosquito-borne diseases need to focus on clearly explaining why these diseases are a severe problem and how they can be controlled or avoided. Any strategy to further enhance individual and collective consciousness and behavior changes must also address the issues associated with poverty in order to achieve a greater impact.^195^

 Further studies on EIDs and RIDs such as MERS, CCHF, AHF, and H5N1 are required to understand better the real epidemiological situation of these diseases in the region. This knowledge, alongside understanding other risk factors, can help reduce the risk of disease spread to humans.

 It should also be noted that in most cases, due to absence of effective therapeutics or vaccines for most EIDs and RIDs, the governmental will to invest in prevention and control measures for these high-threat pathogens may be lacking in some countries in the region. Research is needed to address these critical knowledge gaps in diagnostic, therapeutic, and preventive measures for most EIDs and RIDs in the region.

## Conclusion

 The EMR countries are continuously experiencing large population movements associated with the Hajj and Arba’een pilgrimage; internally displaced populations and refugees; and armed groups and transnational migrants. Furthermore, poverty, climate change, alongside the weak public health infrastructure, has predisposed this region to various EIDs and RIDs. Some diseases are endemic in this region and confer threats to international travellers. Several factors are important for the eradication of infectious diseases in this area, including political will, financial investment, cooperative international and local efforts, massive drug administration, vaccination, and surveillance for detection and diagnoses of lower recognized agents.

 Understanding and documenting the regional scope and epidemiology of these infectious disease outbreaks will contribute greatly to prevent, rapidly identify, and promptly respond to these health threats in order to minimize deaths, limit geographic spread and interrupt transmission using evidence-based and high value interventions. Developing effective evidence-based public health control measures and intervention strategies to minimize the risk of infection is a key priority for countries in the region (Table 2). Research initiatives to learn more about the nature and impact of infectious diseases in the region are needed for better planning and control measures.

**Table 2:** The Major Drivers of Emerging and RIDs and Suggested Control Measures in the WHO EMR

**Disease Name**	**Hepatitis virus A and E**	**Main Vector**	**Main Reservoir**	**Major Risk Factors of Emergence or Re-emergence**	**Suggested Control Measures**	**Reference**
Acute hepatitis A and E	-	-	-	Consumption of sewage-contaminated water; use of indoor dry pits; war and conflict, famine and influx of refugees	Vaccination, providing adequate drinking water and sewage disposal	^31,38^
AHF	AHF virus	Ticks	Camel	Migratory birds, livestock trade, tick infestation; climate change	Tick control; control of livestock trade	^49-51^
AI (H5N1)	Avian influenza virus	-	Migratory bird, Poultry	Migratory birds; local bird trade, war and conflict	One Health strategy, active surveillance, testing and culling the areas where HPAI H5N1 was initially detected	^57,59^
Chikungunya	Chikungunya virus	*Aedes aegypti, Aedes albopictus*	-	Climate change, war and conflict, globalization, the significant increase in international travel and trade, vector resistance to pesticide, lack of competent surveillance system	Mosquito control; Environment hygiene; *Aedes* surveillance, collective consciousness	^13,63,64^
Cholera	*Vibrio cholera*	-	-	Poor living and sanitation; lack of clean water; war and conflict; global warming	Proper sanitation, hygienic life, environment hygiene, early warning system	^140,142,144,146^
CCHF	CCHF virus	Ticks	Livestock	Tick infestation, livestock contact and trading, nosocomial transmission; slaughtering during Eid al-Adha; climate change	Tick control; environment hygiene; education of high-risk population to reduce exposure to the virus	^67,75,76^
Dengue	Dengue virus	*Aedes aegypti, Aedes albopictus*	-	Climate change, war and conflict, globalization, the significant increase in international travel and trade, vector resistance to pesticide, lack of competent surveillance system	Mosquito control; environment hygiene; Aedes surveillance, collective consciousness	^77,79,82,85^
Diphtheria	*Corynebacterium diphtheria*	-	-	Low living condition and sanitation; war and conflict	Vaccination of at-risk population; quick disease diagnosis and management	^146^
Leishmaniasis	*Leishmania infantum, Leishmania tropica, Leishmania killicki*	Sandflies	Rodents and Dogs	War and conflict; poor environment hygiene; massive population displacement; climate change	One Health strategy; vector and reservoir control	^21,176,179,181,196^
Measles	Measles virus	-	-	War and conflict	Vaccination	^87,92^
Meningococcal disease	*Neisseria meningitidis*	-	-	Mass gathering in Hajj and Arba’een	Surveillance and vaccination	^148-150,152^
MERS	MERS-CoV	-	Dromedary camel	Camel contact, consumption of camel products, community contact, nosocomial infection, international travel	One Health strategy, hospital hygiene, farm biosecurity, animal quarantine	^23,98,100,101,108^
Plague	*Yersinia pestis*	Fleas	Rodents, livestock and carnivores	Weakness in the competent surveillance system and laboratory facilities to early diagnose	Monitoring and surveillance; rodent and flea surveillance in high-risk regions	^154-157^
Poliomyelitis	Poliovirus	-	-	War and conflict; weakness in the competent surveillance	Surveillance on all cases of acute flaccid paralysis, vaccination	^136,137,139^
Q fever	*Coxiella burnetii*	Ticks	Livestock	Mass gathering in Hajj and Arba’een	One Health strategy, monitoring and surveillance	^159,160,163,197^
Rabies	Rabies virus	-	Dogs, Foxes and other wild carnivores	War and conflict	One Health strategy, vaccination; reservoir control	^113-117^
RVF	RVF virus	Mosquitoes	Livestock	Mosquito and livestock contact, rainfall; climate change	Mosquito control; environment hygiene; control of livestock trade; animal vaccination, collective consciousness	^119,121,122,124^
Sandfly fever	Sandfly fever virus	Sandflies	-	Climate change; weakness in the competent surveillance system and laboratory facilities	Mosquito control, collective consciousness	^125,126,131^
*Tularemia*	*Francisella tularensis*	Ticks, mosquitoes	Rodents, hares	Weakness in the competent surveillance system and laboratory facilities; war and conflict	Monitoring and surveillance, collective consciousness	^164,165,198^
WNF	WNF virus	Culex mosquito	Birds, equines	Human dwelling at *Culex mosquito* breeding sites; weakness in the competent surveillance system and laboratory facilities	Mosquito control; environment hygiene, collective consciousness	^134,135^

Abbreviations: EMR, Eastern Mediterranean Region; RIDs, re-emerging infectious diseases; WHO, World Health Organization; AHF, Alkhurma hemorrhagic fever; CCHF, Crimean-Congo hemorrhagic fever; MERS-CoV, Middle East respiratory syndrome coronavirus; RVF, Rift Valley fever; WNF, West Nile fever; HPAI, highly pathogenic avian influenza.

 Better targeted investigations should be implemented to identify and prevent widespread emergence and re-emergence of infectious diseases. It is essential to defend the population through multifactorial efforts, including coordinated, well-prepared and well-equipped public health systems alongside partnerships among clinicians, laboratories and public health agencies. Besides, the application of advanced and proper diagnostic methods and surveillance contributes to achieving better results in limited time.

## Acknowledgment

 We would like to thank Dr. Heba Sobhy Ibrahim Mahrous, from WHO for Eastern Meditation region, Cairo, EGYPT, who supported us in improving the first draft of the manuscript.

## Ethical issues

 Not applicable.

## Competing interests

 Authors declare that they have no competing interests.

## Authors’ contributions

 EM carried out the design of the study. AG and SANM participated in gathering the data, and prepared the first draft of manuscript. All authors critically reviewed the manuscript, applied comments and finalized the manuscript.

## Funding

 There is no source of funding for this work.
